# Intake of Phthalate-tainted Foods and Serum Thyroid Hormones in Taiwanese Children and Adolescents

**DOI:** 10.1038/srep30589

**Published:** 2016-07-29

**Authors:** Hui-Ju Tsai, Chia-Fang Wu, Yi-Chun Tsai, Po-Chin Huang, Mei-Lien Chen, Shu-Li Wang, Bai-Hsiun Chen, Chu-Chih Chen, Wen-Chiu Wu, Pi-Shan Hsu, Chao A. Hsiung, Ming-Tsang Wu

**Affiliations:** 1 Department of Family Medicine, Kaohsiung Municipal Ta-Tung Hospital, Kaohsiung Medical University Hospital, Kaohsiung Medical University, Kaohsiung, Taiwan; 2 Department of Public Health, College of Health Sciences, Kaohsiung Medical University, Kaohsiung, Taiwan; 3 Research Center for Environmental Medicine, Kaohsiung Medical University, Kaohsiung, Taiwan; 4 Division of Nephrology, Department of Internal Medicine, Kaohsiung Medical University Hospital, Kaohsiung Medical University, Kaohsiung, Taiwan; 5 National Environmental Health Research Center, National Health Research Institutes, Miaoli, Taiwan; 6 Institute of Environmental and Occupational Health Sciences, College of Medicine, National Yang Ming University, Taipei, Taiwan; 7 National Environmental Health Research Center, National Institute of Environmental Health Sciences, National Health Research Institutes, Miaoli, Taiwan; 8 Department of Laboratory Medicine and Pediatrics, Kaohsiung Medical University Hospital, Kaohsiung Medical University, Kaohsiung, Taiwan; 9 Graduate Institute of Clinical Medicine, College of Medicine, Kaohsiung Medical University, Kaohsiung, Taiwan; 10 Division of Biostatistics and Bioinformatics, Institute of Population Health Sciences, National Health Research Institutes, Miaoli, Taiwan; 11 Department of Pediatrics, Taipei Hospital, Ministry of Health and Welfare, Taipei, Taiwan; 12 Department of Family Medicine, Taichung Hospital, Ministry of Health and Welfare, Taichung; 13 Department of Family Medicine, Kaohsiung Medical University Hospital, Kaohsiung Medical University, Kaohsiung, Taiwan

## Abstract

On April-May, 2011, phthalates, mainly Di-(2-ethylhexyl) phthalate (DEHP), were deliberately added to a variety of foodstuff as a substitute emulsifier in Taiwan. This study investigated the relationship between DEHP-tainted foodstuffs exposure and thyroid function in possibly affected children and adolescents. Two hundred fifty participants <18 years possibly exposed to DEHP were enrolled in this study between August 2012 and January 2013. Questionnaires were used to collect details on their past exposure to DEHP-tainted food items. Blood and urine samples were collected for biochemical workups to measure current exposure derived from three urinary DEHP metabolites using a creatinine excretion-based model. More than half of 250 participants were estimated to be exposed to DEHP-tainted foods found to exceed the recommend tolerable daily intake of DEHP established by the European Food Safety Authority (<50 μg/kg/day). The median daily DEHP intake (DDI) among those 250 participants was 46.52 μg/kg/day after multiple imputation. This value was ~10-fold higher than the current median DEHP intake (4.46 μg/kg/day, n = 240). Neither past nor current DEHP exposure intensity was significantly associated with serum thyroid profiles. Future studies may want to follow the long-term health effects of this food scandal in affected children and adolescents.

April–May, 2011, a major health scandal involving phthalate-tainted foodstuffs came to light in Taiwan^1^. Phthalates, mainly DEHP (Di-(2-ethylhexyl) phthalate) and DINP (Di-isononyl phthalate), were deliberately added to a variety of foodstuffs as a substitute emulsifier. Five categories of foodstuff, including sports drinks, fruit beverages, tea drinks, fruit, jam or jelly, and health foods or supplements in tablet or powder form, were contaminated by those two phthalate chemicals. The food items categorized as “health food or supplements in tablet, capsule or powder form” are commonly consumed by children there^1^. Based on the data from the official website of Taiwan’s Food and Drug Administration (TFDA), DEHP-contaminated concentrations were highest in food items in that particular category, where levels reached as high as 2,108.0 ppm^1^.

Phthalates are thought to affect endocrine functions including thyroid profiles^2^,^3^,^4^,^5^. Our previous study found that serum thyroid-stimulating hormone (TSH) levels were significantly and negatively associated with daily intake of DEHP-tainted foods (Spearman correlation coefficient r = 0.422, P = 0.0048) in the 60 potentially exposed children aged ≤10 yrs recruited from one hospital in Kaohsiung city located in southern Taiwan^6^.

The companies involved in this food scandal were transnational, and thus some of the phthalate-tainted food items (206 food products from 34 manufacturers) had been exported to as many as twenty-two countries, including the USA and the European Union^1^. Because this deliberate use of phthalates in food manufacturing could potentially influence the health of all people, particularly children and adolescents, the Taiwan government requested Taiwan’s National Health Research Institutes (NHRI), to administer a nationwide health survey to people potentially exposed to phthalate-tainted foods^7^. The survey included intake of food items whose DEHP concentrations were known. Since that nationwide survey was more representative of the whole population than our previous area specific survey^6^, we re-examined the impact of past intake of phthalate-tainted foods, particularly those contaminated by DEHP, on thyroid hormones in potentially affected children and adolescents (<18 yrs). We hypothesized that thyroid function in children and adolescents could be affected by exposure of DEHP-tainted food items and that the impact could be either reversible or irreversible. At the time of the survey, we also measured the metabolites of several phthalate chemicals in the urine of the children and adolescents taking the questionnaire survey, and thus we were additionally able to examine the effect of current exposure based on urinary DEHP’s oxidative metabolites and metabolites from other phthalates on changes in thyroid hormones.

## Result

**Figure 1:**
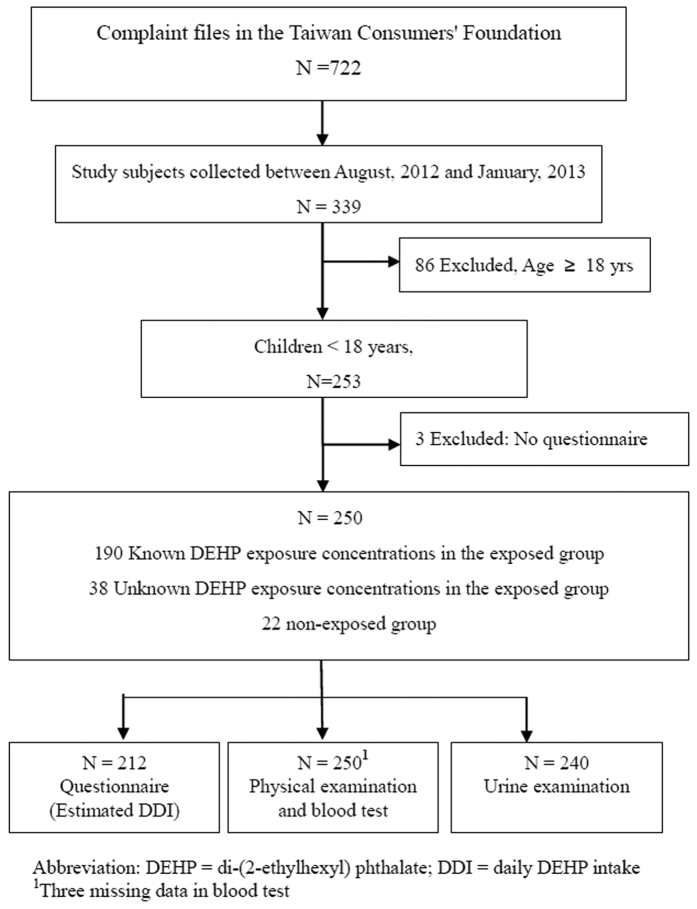
Study flowchart.

Seven hundred and twenty-two subjects complained to the Taiwan Consumers’ Foundation. Of these, 339 (47.0%) sought medical consultation at one of three special phthalates centers and expressed willingness to participate in this study between August, 2012 and January, 2013 (Fig. 1). Of those participants, 253 were aged <18 yrs. Three of them were excluded because their questionnaires were not complete. The remaining 250 eligible study children and adolescents were healthy.

Table 1 shows the distribution of past and current DEHP exposure intensity, urinary phthalate metabolites, and serum thyroid profile before and after multiple imputations. After multiple imputation, the median daily DEHP intake (DDI) of 250 study children or adolescents was 46.52 μg/kg/day. This value was ~10-fold higher than the current median intake estimated from urine DEHP metabolites (4.46 μg/kg/day, n = 240) (Table 1). In general, the exposure data did not change dramatically before and after imputations.

Categorized by the recommended tolerable daily intakes (TDI) of DEHP established by the European Food Safety Authority (EFSA) (TDI < 50 μg/kg/day)^8^ and U.S. Environmental Protection Agency (USEPA) (TDI < 20 μg/kg/day)^9^, we found that 117 and 181 of 250 participants (46.8% and 72.4% respectively) had been exposed to DEHP-tainted foods exceeding the TDI of EFSA and USEPA, respectively, after multiple imputation (Table 2). Before the multiple imputation, results were similar showing that more than half (60.4%) of the participants (128/212) were exposed to DEHP-tainted foods exceeding the TDI of EFSA (see Supplementary Table S1).

Multivariate linear regression models did not reveal any significant difference between the past DEHP exposure intensity and any serum marker of thyroid function (Table 3). The results remained non-significant when examining the effect of current DEHP exposure estimated by creatinine excretion-based model derived from urinary mono-(2-ethylhexyl) phthalate (MEHP). mono (2-ethyl-5-hydroxyhexyl) phthalate (MEHHP), and mono-(2-ethyl-5-oxohexyl) phthalate (MEOHP) on thyroid functions (Table 3).

Table 4 shows the association between each urinary phthalate metabolite and serum thyroid functions. Using the *p-*values corrected by Benjamini-Hochberg method, the only associations we found to be significant was between both urine mono-n-butyl phthalate (MnBP) and mono-benzyl phthalate (MBzP) and serum TSH level reached (both *p-*values = 0.04). However, both adjusted β estimates were relatively small (both β = 0.001). In the sensitivity analysis, similar non-significant results were also found after excluding 22 participants as non-exposed before multiple imputation (see Supplementary Tables S2 and S3).

## Discussion

This study found no significant association between past DEHP exposure based on past intake of DEHP-tainted foodstuff or current DEHP exposure estimated using urinary DEHP metabolites and the levels of any of thyroid hormones we studied. Of the urinary phthalate metabolites, only MnBP and MBzP were significantly associated with serum TSH level after multiple imputation, though the effect was small and the significances were absent in the sensitivity analysis.

When the phthalate food scandal came to light in 2011, we immediately enrolled 60 affected children ≤10 yrs old from one medical center located in southern Taiwan for our previous study surveying the health consequences of this scandal between May 31 and June 2011^6^. That study found that serum TSH levels were significantly and negatively associated with daily intake of phthalate-tainted foods in children, and it found that triiodothyronine (T3) may be partially recovered after stopping exposure^6^. The current study found no significant association between the two. The reasons for this inconsistency in finding are unclear. We collected serum for the thyroid profiling at least one year after the food scandal occurred (between August, 2012 and January, 2013), thus, thyroid hormone levels may have recovered after ceasing intake of phthalate-tainted foods. In addition, the study participants in the present study were from all parts of Taiwan, but study participants in the previous study were residents of one specific area, Kaohsiung City. We re-checked the study subjects in the two studies and only found two children who had participated in both studies. A further study may be needed to explore the differences in the findings of these studies.

According to animal studies, phthalates may disrupt thyroid function through different pathways: (1) they could affect the T3 bindings to transport proteins; (2) they might interact with the uptake of active T3 in plasma membrane; (3) they might act as an antagonist at the thyroid hormone receptors; and (4) they might affect the transcriptional activity of sodium/iodide symporter or thyroid hormone receptors, etc^10^,^11^,^12^,^13^,^14^. Animal studies have shown that male rats exposed to the relatively high concentration of 10,000 ppm DEHP for 14 days had a reduction in follicular size and morphological changes in follicular cells in the thyroid gland^15^,^16^. More research is needed to explore specific mechanisms through which environmental phthalate exposure levels alter thyroid function and cause morphological change in murine models.

A series of human studies have investigated the relationship between phthalate exposure and serum thyroid functions in adults and children and their findings were reviewed in our previous study^17^,^18^,^19^,^20^. Briefly, Meeker *et al*. first analyzed DEHP metabolites, including MEHP, MEHHP, and MEOHP in one-spot urine and serum TSH, free thyroxine (FT4) and T3 in 408 men in one Fertility Center in Massachusetts, USA^17^. Their results revealed a significant inverse association between MEHP and serum FT4 levels. In that study, MEHHP was significantly and positively associated with FT4 in a subgroup of 208 men. Meeker & Ferguson, tapping data collected by the 2007–2008 National Health and Nutrition Examination Survey (NHANES), studied 1,346 adults aged ≥20 years and 329 adolescents aged 12–19 years^19^. They observed that only MEHHP of DEHP metabolites had monotonic dose-dependent decreases in thyroxine (T4). In adolescents, they found a significant and positive association between secondary DEHP metabolites and T3 and TSH. Another cross-sectional study of 845 Danish children aged 4–9 years showed the sum of urinary DEHP metabolites were negatively associated with serum free triiodothyronine (FT3) level^18^. Although these studies supposed that certain phthalates may disrupt thyroid function in adults and children, they were not able to conclude which phthalates altered which specific thyroid hormones and how long the effects of phthalates could last on thyroid functions. Most studies tried to link the phthalates concentrations in one-spot urine with specific health outcomes of interest. It is doubtful that causality between exposure and outcome of interests can be clearly established.

We found that the median daily DEHP intake among the 250 study children and adolescents was 46.52 μg/kg/day, which was ~10-fold higher than the current median intake estimated from urine DEHP metabolites (4.46 μg/kg/day, n = 240). The half-life of phthalates, including DEHP, is very short, less than several days. This finding was also demonstrated in our previous study^21^. At 6-month follow-up, all DEHP metabolite concentrations in urine of the study children were significantly decreased compared to baseline^21^. This may also explain the reason that we did not find a good correlation between the past DEHP exposure estimated by questionnaire and the current DEHP exposure calculated by creatinine excretion-based model.

Most previous studies have measured urine phthalate metabolites in one-spot urine samples by correcting urine creatinine to take into account the urine dilution effect and better represent external environmental DEHP exposure^21^,^22^. However, one recent study performed by O’Brien *et al*. suggested that the new method of covariate-adjusted standardization plus creatinine adjustment to correct not only the urine concentration but also the complicated confounding structure is more representative for the accurate environmental phthalate exposure^22^. We performed both analyses and found the results to remain similar.

Although the sample size in this study was much larger than that of our previous study^6^, it has some limitations. One limitation is that there was a time lag of at least one year due to the delay in Institutional Review Boards (IRBs) approvals from different cooperating institutions^1^. This time lag may have resulted in some recall bias with regard to reported intake. Another limitation is that there is a possibility of volunteer bias because the study children were recruited from a group of people who filed complaints. Still another limitation is that we did not measure thyroid profiles when the incident was first made public. Therefore, changes in thyroid function could not be compared. Further, because we did not have the authority to gather personal information such as age and gender among those non-participants, we were unable to compare the differences between participants and non-participants. Finally, we did not focus much on the effects of DINP-tainted foods because DEHP has a higher toxicity than DINP and because there was less information about DINP concentrations of DINP in this food scandal.

We conclude that the estimated past and present DEHP exposure levels were not significantly associated with any thyroid function markers in serum. A follow-up study of these affected children and adolescents is necessary to evaluate the long-term health consequences of this food scandal.

## Materials and Methods

### Study subjects

This study was first approved by the IRBs of NHRI, the Ministry of Health and Welfare Hospitals (MHWHs), and Kaohsiung Medical University Hospital (KMUH)^7^. The reference number of the approvals was NHRI-EC100090 and KMUH-IRB-2012-11-01. The methods were carried out in accordance with the approved guidelines.

Potential participants were recruited from people who came to the Taiwan Consumers’ Foundation to complain they were possible victims of the phthalate-tainted foods and filed a lawsuit for compensation. Those who filed complaints were encouraged by the officials from Taiwan Food and Drug Administration (TFDA), Ministry of Health and Welfare, to receive medical consultation. Those wanting medical consultation were transferred to one of three phthalates clinics established in different hospitals, including the MHWHs in Taipei City and in Taichung City as well as KMUH in Kaohsiung City, located in northern, central, and southern Taiwan, respectively, between August, 2012 and January, 2013^7^. The current study focused on the children and adolescents aged <18 yrs whose parents were willing to participate in this study. All participants provided written informed consent.

### Past exposure based on questionnaire reported intake of phthalate-contaminated food items

All study children and adolescents received physical examinations performed by pediatricians. The detailed questionnaire created to gather information on intake of phthalates-tainted foodstuff has been described previously^6^,^7^. Using this standardized questionnaire, three trained interviewers questioned the main caregivers, mostly mothers, of the study children and adolescents about their children’s and adolescents’ food intake. Any foodstuff on the questionnaire found to have DEHP or DINP ≥1 ppm was defined as a phthalates-tainted foodstuff. DEHP concentrations of these food items were extracted from two sources: TFDA and the Bureau of Health of Kaohsiung City (KBOH)^6^,^7^,^21^.

Using the intake information gathered from the questionnaire and the published known DEHP concentrations in phthalate-tainted foods, we constructed a daily DEHP intake (DDI, μg/kg body weight (bw)/day) of the phthalate-tainted foodstuffs^6^,^7^,^21^. DDI was based on the exposure amount (μg per time) and frequency (times per day) divided by body weight (kg) of each participant. Those children and adolescents not exposed to any listed phthalate-tainted food items were categorized into non-exposed group^6^,^7^,^21^. Our previous study found that the combined intake information from the questionnaire and two governmental reports of DEHP concentrations correlated well with DEHP metabolites in urine collected from 23 children during the phthalate scandal (Spearman correlation r = 0.466, p = 0.025)^21^.

### Current exposure based on measurement of phthalate metabolites in urine

The children and adolescents provided one-spot urine samples after receiving physical examinations and being interviewed with the questionnaire in the special phthalates clinic in each hospital^7^. The urine samples were aliquoted and stored in a −20° C freezer until measurement of phthalate metabolites.

We measured nine phthalate metabolites in the urine samples. The metabolites, which were mono-methyl phthalate (MMP), mono-ethyl phthalate (MEP), MnBP, MBzP, MEHP, MEOHP, MEHHP, mono-isobutyl phthalate (MiBP) and monoisononyl phthalate (MiNP), represented the seven most commonly used phthalates (DEHP, DnBP, DiBP, BBzP, DMP, DEP, and DINP) in the environment. The analytical methods have been described in detail previously^7^,^23^,^24^. Briefly, the nine urinary phthalate metabolites were analyzed by online solid phase extraction (SPE), coupled with liquid chromatography/electrospray ionization tandem mass spectrometry (LC-ESI-MS/MS) in a central NHRI analytical laboratory, which had been certified by an international laboratory comparison program (G-EQUAS 52).

The method of detection limits (MDL) for MMP, MEP, MnBP, MiBP, MBzP, MEHP, MEHHP, MEOHP, and MiNP determined using a urine sample spiked with standard, were 0.12, 0.12, 0.12, 0.24, 0.12, 0.24, 0.12, 0.24, and 1.20 ng/mL respectively. A measurement below MDL was treated as half of a MDL. Because MiNP was detectable in only four of 240 urine samples, we only presented the findings for the remaining eight urinary phthalate metabolites.

To more accurately estimate environmental phthalate exposure by using the measurement of phthalate metabolites from one-spot urine sample, we adopted a new method of covariate-adjusted standardization plus creatinine adjustment to correct for urine concentration and complicated confounding structure, as suggested by O’Brien *et al*.^22^.

### Current estimated DEHP daily intake based on urinary DEHP metabolites

Current daily DEHP intake in each study child or adolescent was also estimated based on a creatinine excretion-based model from the urinary DEHP metabolite including MEHP, MEHHP and MEOHP. How this value was calculated has been described in detail previously^21^,^25^,^26^,^27^,^28^. The creatinine excretion-based model followed the equation: DEHP (μg/kg/day) = [UE_sum_(μmol/g creatinine) × CE_smoothed_ (g creatinine/day)/F_UE _× bw (kg)] × MW_DEHP_, where UE_sum_ (μmol/g creatinine) was the molar urinary excretion sum of MEHP, MEOHP, and MEHHP adjusted for creatinine. CE_smoothed_ (g creatinine/day) was the daily creatinine excretion rate according to the study of Remer *et al*.^27^. MW represented the molecular weight of a given DEHP metabolite. F_UE_ (44.2%) was the sum of the proportions of MEHP, MEHHP, and MEOHP excreted in urine following the ingested DEHP^28^.

### Measurement of endocrine hormones in serum

Blood samples were collected by phlebotomy after the children had fasted for twelve hours and were immediately centrifuged at 4 °C for 20 min. The supernatant of serum was aliquoted and stored at −80 °C until analysis. The endocrine profile included TSH, T4, FT4, and T3, all measured by chemiluminescence immunoassay in a central clinical laboratory in Taipei. This laboratory is officially accredited by Taiwan Accreditation Foundation based on the accreditation criteria of ISO 15189:2007 and the effective period of this certificate (Certificate No. L1447–120220) was between 16 May 2012 and 15 May 2015. The analytical sensitivity of chemiluminescence immunoassay was 0.001μU/mL for TSH, 0.3 μg/dL for T4, 0.1 ng/dL for FT4, and 10.0 ng/dL for T3. All serum thyroid hormone levels in the children were detectable.

### Statistical analysis

Our main research goal was to examine whether past exposure to DEHP-tainted foodstuffs estimated by questionnaire or current DEHP exposure estimated by creatinine excretion-based model affected the thyroid profiles in children and adolescents exposed to tainted food items. All statistical operations were performed using SPSS version 18 and SAS 9.3. The *p-*values were two-sided and considered significant if < 0.05. In addition to measuring urine DEHP metabolites, we also measured the metabolites of DnBP, DiBP, BBzP, DMP, DEP, and DINP in urine. To account for the multiple comparisons of phthalate metabolites (n = 8), we used Benjamini-Hochberg method to correct *p-*values^29^.

Approximately 15.2% (38 out of 250) study children or adolescents were known to be exposed to phthalate-tainted foodstuffs, but we were not able to access their exact DEHP concentration levels. In addition, another 22 study children or adolescents were not found to be exposed to any listed DEHP-tainted foodstuffs, based on the above-mentioned two sources: TFDA and KBOH. To avoid the exclusion of informative missing data and the misclassified exposure levels due to the limited phthalate-tainted food sources of TFDA and KBOH, we used the multiple imputation method to assign those 60 study children and adolescents into the DEHP exposure levels from phthalate-tainted foods^30^,^31^. Multiple imputation with four imputations for those samples were performed following standard rules described in a study by Rubin to achieve 96% to 97% relative efficiency to ensure in-range values^30^. A similar imputation approach was also applied to other covariates with missing data.

First, demographic characteristics and thyroid hormone levels were compared by Kruskal-Wallis test or Fischer’s exact test among different exposure groups categorized by TDI of USEPA (<20 μg/kg/day) and EFSA (< 50 μg/kg/day) before and after multiple imputations. Then, multivariate linear regression model was used to analyze the association between either past DEHP exposure or current DEHP exposure and thyroid profiles after adjusting for the covariates. Since all serum markers of thyroid function were normally distributed, we did not transform them in the models. Exposure of interest (past DEHP exposure intensity or current DEHP exposure intensity) was treated as continuous variable and into quartiles, producing similar results. Thus, we only presented it as a continuous variable. The results of adjustments for other covariates were similar to those obtained when adjusting for age, gender, and body mass index (BMI), and thus we only presented findings adjusted for age, gender, and BMI. The same analytical strategies were also applied to examine the association between eight individual phthalate metabolites and thyroid profiles.

### Sensitivity analysis

To check the robustness of the analyses after multiple imputation, we excluded those twenty-two study children or adolescents not exposed to any of listed phthalate-tainted food items based on two sources of TFDA and KBOH and reran the statistical analyses.

## Additional Information

**How to cite this article**: Tsai, H.-J. *et al*. Intake of Phthalate-tainted Foods and Serum Thyroid Hormones in Taiwanese Children and Adolescents. *Sci. Rep.* **6**, 30589; doi: 10.1038/srep30589 (2016).

## Supplementary Material

Supplementary Information

## Figures and Tables

**Table 1:** Distribution of estimated DEHP intake, urinary hthalate metabolites, and serum thyroid profiles in study children.

Variables	N	Percentiles						
		Min	5th	25th	50th	75th	95th	Max
Daily DEHP intake (DDI) estimated by questionnaire (μg/kg/day)								
All study children before multiple imputation^1^	212	0	0	2.72	31.41	59.68	155.79	705.34
All study children after multiple imputation^2^	250	0.00001	0.65	16.46	46.52	65.23	154.08	705.34
Children with intake of DEHP-tainted foods^3^	190	0.23	0.72	11.00	38.81	64.97	156.32	705.34
Children with intake of DEHP-tainted foods after multiple imputation^4^	228	0.00001	0.68	14.76	44.24	63.40	155.32	705.34
Daily DEHP intake (DDI) estimated by creatinine excretion-based model (μg/kg/day)								
All study children^5^	240	0.20	1.55	3.05	4.46	6.86	16.06	40.46
Urine^5^								
Urinary creatinine (mg/dL)	240	5.00	13.85	29.88	47.95	78.95	138.68	255.50
Before the correction of urine creatinine								
MMP (μg/L)	240	0.06	0.94	3.63	6.39	13.63	34.46	161.78
MEP (μg/L)	240	0.04	0.15	2.89	7.29	14.45	73.73	318.94
MnBP (μg/L)	240	1.68	4.13	10.37	21.39	44.37	101.23	229.12
MBzP (μg/L)	240	0.06	0.06	0.73	1.34	2.84	8.80	194.21
MEHP (μg/L)	240	0.12	0.12	1.90	4.77	9.78	23.43	67.54
MEHHP (μg/L)	240	0.62	3.88	10.29	20.62	35.91	113.65	366.71
MEOHP (μg/L)	240	0.05	2.70	7.27	14.70	26.86	74.79	185.33
MiBP (μg/L)	240	0.12	2.34	5.44	9.80	22.58	67.90	546.95
After the correction of urine creatinine								
MMP (μg/g Cr)	240	0.33	11.49	31.74	54.88	105.29	246.02	2344.55
MEP (μg/g Cr)	240	0.47	1.30	24.72	53.16	108.40	402.72	2086.47
MnBP (μg/g Cr)	240	21.77	58.21	108.83	172.55	278.89	597.26	1278.80
MBzP (μg/g Cr)	240	0.25	0.47	6.34	10.81	21.08	62.48	2719.35
MEHP (μg/g Cr)	240	0.73	2.30	19.24	36.29	64.62	133.03	387.36
MEHHP (μg/g Cr)	240	6.62	50.95	114.30	166.48	238.30	566.02	1569.36
MEOHP (μg/g Cr)	240	0.41	35.37	80.75	119.00	170.48	392.71	1030.17
MiBP (μg/g Cr)	240	8.25	26.21	58.69	92.27	152.71	299.92	2610.56
Serum thyroid profile^6^								
T3 (ng/dl)	247	83.00	102.80	124.00	140.00	158.00	184.20	228.00
T4 (μg/dl)	247	5.30	6.94	8.60	9.70	10.80	11.96	13.70
FT4 (ng/dl)	247	0.88	0.99	1.12	1.20	1.28	1.46	1.69
TSH (μIU/ml)	247	0.26	0.93	1.57	2.29	3.05	4.76	6.99

Abbreviations: Cr = creatinine; DDI = daily DEHP intake; DEHP = di-(2-ethylhexyl) phthalate; FT4 = free thyroxine; Max = maximum; Min = minimum; MEHP = mono-(2-ethylhexyl)phthalate; MEOHP = mono-(2-ethyl-5-hydroxylhexyl) phthalate; MEHHP = mono-(2-ethyl-5-oxohexyl) phthalate; MiBP = mono-iso-butyl phthalate; MnBP = mono-n-butyl phthalate; MBzP = mono-benzyl phthalate; MEP = mono-ethyl phthalate; MMP = mono-methyl phthalate; MiNP = mono-isononyl phthalate; T3 = triiodothyronine; T4 = thyroxine; TSH = thyroid-stimulating hormone.

^1^Participants with known DEHP exposure concentrations from questionnaire for the estimate of DDI before multiple imputation.

^2^Multiple imputation for 38 missing data in the exposed group and 22 no-exposed group.

^3^Excluding 22 non-exposed group before multiple imputation.

^4^Excluding 22 non-exposed group after multiple imputation.

^5^Ten missing data without adequate urine samples for analysis.

^6^Three missing data in blood test.

**Table 2:** Characteristics and Clinical Findings of Study Children and Adolescents Categorized by Tolerable Daily Intake (TDI) of US Environmental Protection Agency and European Food Safety Authority After Multiple Imputation (N = 250)^1^.

Category (μg/kg/day)	>50 N = 117	≤50, >20 N = 64	≤20 N = 69	P Value^2^
DDI (μg/kg/day)	87.1 ± 69.6 (66.2, 57.0–84.5)	36.0 ± 8.6 (36.0, 28.2–43.2)	5.6 ± 6.1 (2.5, 1.1–10.6)	
Mean ± SD (Median, IQR) or N (%)				
Age (yrs)	4.5 ± 2.0 (4.0, 3.0–6.0)	5.9 ± 2.5 (6.0, 4.0–7.0)	8.3 ± 3.7 (7.0, 5.5–10.5)	<0.001
Gender				
Female	42 (35.9)	34 (53.1)	28 (40.6)	0.08
Male	75 (64.1)	30 (46.9)	41 (59.4)	
Height (cm)	107.2 ± 13.0 (106.4, 98.7–114.2)	116.1 ± 14.5 (116.8, 106.4–125.7)	131.9 ± 21.9 (131.5, 116.1–147.3)	<0.001
Weight (kg)	18.2 ± 5.2 (17.5, 15.0–20.7)	22.2 ± 6.4 (21.0, 17.5–26.0)	33.8 ± 15.9 (28.7, 22.1–42.3)	<0.001
BMI (kg/m^2^)	15.6 ± 1.5 (15.4, 14.6–16.7)	16.2 ± 2.2 (15.8, 15.0–16.8)	18.3 ± 3.3 (17.4, 15.7–20.2)	<0.001
Endocrine findings				
T3 (ng/dL)	145.8 ± 25.9 (146.0, 126.0–163.0)^3^	139.2 ± 23.9 (136.5, 122.5–153.8)	134.2 ± 23.7 (132.0, 121.0–149.5)^4^	0.01
T4 (μg/dL)	9.8 ± 1.6 (9.9, 8.6–10.8)^3^	9.6 ± 1.4 (9.6, 8.6–10.7)	9.2 ± 1.7 (9.3, 8.3–10.5)^4^	0.14
FT4 (ng/dL)	1.2 ± 0.1 (1.2, 1.1–1.3)^3^	1.2 ± 0.1 (1.2, 1.1–1.3)	1.2 ± 0.2 (1.2, 1.1–1.3)^4^	0.19
TSH (μU/mL)	2.6 ± 1.2 (2.4, 1.8–3.1)^3^	2.3 ± 1.2 (1.9, 1.4–3.1)	2.3 ± 1.1 (2.2, 1.5–3.0)^4^	0.12
	**N = 110**	**N = 63**	**N = 67**	
Urinary metabolites				
MEHP (μg/g Cr)	58.5 ± 59.8 (41.2, 21.7–85.0)	43.5 ± 47.0 (27.2, 12.8-–59.4)	37.6 ± 25.7 (32.9, 17.5–50.7)	0.05
MEOHP (μg/g Cr)	177.9 ± 151.2 (134.5, 92.1–205.3)	143.0 ± 117.0 (106.4, 82.0–170.5)	107.0 ± 61.4 (103.7, 70.3–134.5)	0.002
MEHHP (μg/g Cr)	257.1 ± 241.6 (195.5, 125.4–283.7)	199.0 ± 169.6 (146.3, 114.2–225.8)	160.3 ± 103.5 (150.5, 97.0–201.3)	0.001
DEHP intake estimated by creatinine excretion-based model (μg/kg/day)	6.9 ± 6.2 (5.1, 3.5–8.1)	5.3 ± 3.7 (4.3, 3.1–6.4)	4.3 ± 2.7 (3.6, 2.5–5.4)	0.001

Abbreviations: BMI = body mass index; Cr = creatinine; DDI = daily DEHP intake; DEHP = di-(2-ethylhexyl) phthalate; FT4 = free thyroxine; IQR = interquartile range; MEHP = mono-(2-ethylhexyl) phthalate; MEOHP = mono-(2-ethyl-5-hydroxylhexyl) phthalate; MEHHP = mono-(2-ethyl-5-oxohexyl) phthalate; T3 = triiodothyronine; T4 = thyroxine; TSH = thyroid-stimulating hormone.

^1^Multiple imputation for missing data in the exposed group (n = 38) and no-exposed group (n = 22).

^2^Kruskal-Wallis test for continuous variables and Fischer’s exact test for category variables.

^3^Two missing data.

^4^One missing data.

**Table 3:** Relationship between serum thyroid profiles and di-(2-ethylhexyl) phthalate (DEHP) intake estimated by questionnaire and DEHP estimates by creatinine excretion-based model in multivariate linear regression models after multiple imputation.

	Crude					Adjusted Model^1^			
	N	β	SE	*P*	P*	β	SE	*P*	P*
T3									
Daily DEHP intake (DDI) estimated by questionnaire	250	0.07	0.03	0.01	0.04	0.04	0.03	0.11	0.23
DEHP intake estimated by creatinine excretion-based model^2^	240	0.15	0.32	0.64	0.64	0.05	0.29	0.86	0.86
T4									
Daily DEHP intake (DDI) estimated by questionnaire	250	0.001	0.002	0.65	0.76	−0.002	0.002	0.38	0.51
DEHP intake estimated by creatinine excretion-based model^2^	240	−0.01	0.02	0.64	0.64	−0.02	0.02	0.27	0.54
FT4									
Daily DEHP intake (DDI) estimated by questionnaire	250	0.00005	0	0.76	0.76	0.00006	0	0.72	0.72
DEHP intake estimated by creatinine excretion-based model^2^	240	0.002	0.002	0.39	0.64	0.001	0.002	0.46	0.61
TSH									
Daily DEHP intake (DDI) estimated by questionnaire	250	0.003	0.001	0.04	0.07	0.002	0.001	0.09	0.23
DEHP intake estimated by creatinine excretion-based model^2^	240	0.04	0.02	0.01	0.04	0.04	0.02	0.02	0.08

Abbreviations: BMI = body mass index; DDI = daily DEHP intake; DEHP = di-(2-ethylhexyl) phthalate; FT4 = free thyroxine; T3 = triiodothyronine; T4 = thyroxine; TSH = thyroid-stimulating hormone.

^*^Benjamini-Hochberg adjusted P values.

^1^Adjusting for age, gender, BMI.

^2^Ten missing data.

**Table 4:** Relationship between serum thyroid profiles and urinary phthalate metabolites in multivariate linear regression models (N = 240).

	T3						T4						FT4						TSH					
	Crude			Adjusted^1^			Crude			Adjusted^1^			Crude			Adjusted^1^			Crude			Adjusted^1^		
MMP	−0.01(0.01)	0.52	0.59	−0.003(0.007)	0.71	0.81	0(0.001)	0.56	0.74	0(0.001)	0.42	0.56	0.00005(0)	0.31	0.63	0.00005(0)	0.28	0.59	0(0)	0.73	0.73	0.0001(0)	0.81	0.81
MEP	0.02(0.01)	0.03	0.16	0.01(0.01)	0.12	0.75	−0.001(0)	0.25	0.74	−0.001(0)	0.12	0.35	0.00004(0)	0.40	0.63	0.00003(0)	0.5	0.59	0(0)	0.31	0.35	0(0)	0.40	0.45
MnBP	0.02(0.01)	0.08	0.16	0.01(0.01)	0.37	0.75	0(0.001)	0.48	0.74	−0.001(0.001)	0.17	0.35	0(0)	0.01	0.10	0(0)	0.01	0.06	0.001(0)	0.004	0.03	0.001(0)	0.01	0.04
MBzP	−0.01(0.01)	0.33	0.44	−0.003(0.01)	0.71	0.81	−0.001(0.001)	0.09	0.73	0(0.001)	0.38	0.56	0.00001(0)	0.80	0.80	0.00002(0)	0.70	0.70	0.001(0)	0.03	0.05	0.001(0)	0.01	0.04
MEHP	0.02(0.03)	0.61	0.61	0.01(0.03)	0.65	0.81	0.001(0.002)	0.50	0.74	0.001(0.002)	0.69	0.79	0(0)	0.33	0.63	0(0)	0.37	0.59	0.002(0.002)	0.16	0.21	0.002(0.002)	0.21	0.29
MEHHP	0.01(0.01)	0.26	0.42	0.00002(0.01)	0.99	0.99	0(0.001)	0.65	0.74	−0.001(0.001)	0.18	0.35	0.00003(0)	0.58	0.66	0.00003(0)	0.50	0.59	0.001(0)	0.02	0.05	0.001(0)	0.05	0.10
MEOHP	0.03(0.01)	0.04	0.16	0.01(0.01)	0.38	0.75	0(0.001)	0.58	0.74	−0.001(0.001)	0.11	0.35	0.00004(0)	0.57	0.66	0.00005(0)	0.48	0.59	0.001(0.001)	0.02	0.05	0.001(0.001)	0.05	0.10
MiBP	0.01(0.01)	0.08	0.16	0.01(0.01)	0.21	0.75	0.00005(0)	0.92	0.92	−0.0001(0)	0.84	0.84	0.00008(0)	0.06	0.24	0.00009(0)	0.03	0.12	0.001(0)	0.10	0.15	0.001(0)	0.11	0.17

Abbreviations: BMI = body mass index; FT4 = free thyroxine; MBzP = mono-benzyl phthalate; MEHHP = mono(2-ethyl-5-hydroxyhexyl) phthalate; MEHP = mono-(2-ethylhexyl) phthalate; MEOHP = mono-(2-ethyl-5-oxohexyl) phthalate; MEP = mono-ethyl phthalate; MMP = mono-methyl phthalate; MiBP = mono-isobutyl phthalate; MnBP = mono-n-butyl phthalate; T3 = triiodothyronine; T4 = thyroxine; TSH = thyroid-stimulating hormone.

^*^Benjamini-Hochberg adjusted P values.

^1^Adjusted for age, gender, and BMI.
