# Supplementary material for: Intake of Phthalate-tainted Foods and Serum Thyroid Hormones in Taiwanese Children and Adolescents
Source: Sci Rep. 2016 Jul 29;6:30589. doi: 10.1038/srep30589 (PMC4965773; doi:10.1038/srep30589)
Supplement: Supplementary Information [file srep30589-s1.pdf]

Supplementary Information

Intake of Phthalate-tainted Foods and Serum Thyroid Hormones in Taiwanese Children and Adolescents

Hui-Ju Tsai, Chia-Fang Wu, Yi-Chun Tsai, Po-Chin Huang, Mei-Lien Chen, Shu-Li Wang, Bai-Hsiun Chen, Chu-Chih Chen, Wen-Chiu Wu, Pi-Shan Hsu, Chao A. Hsiung, Ming-Tsang Wu

Table S1. Characteristics and Clinical Findings of Study Children and Adolescents Categorized by Tolerable Daily Intake (TDI) of U.S. Environmental Protection Agency and European Food Safety Authority before multiple imputation (N=212).[1]

**Category (μg/kg/day)**	**> 50 N=76**	**≤ 50, > 20 N=52**	**≤ 20 N=84**	**P Value[2]**
DDI (μg/kg/day)	100.6 ± 83.3 (75.5, 57.0-121.0)	34.6± 8.5 (34.5, 27.0-42.5)	4.3 ± 5.8 (1.7, 0.0-6.4)	
		Mean ± SD (Median, IQR) or N (%)		
Age (yrs)	4.6 ± 2.2 (4.0, 3.0-6.0)	5.4 ± 2.5 (5.0, 3.8-7.0)	7.3 ± 3.7 (6.5, 4.0-10.0)	<0.001
Gender				
Female	29 (38.2)	26 (50.0)	33 (39.3)	0.37
Male	47 (61.8)	26 (50.0)	51 (60.7)	
Height (cm)	108.2 ± 14.2 (106.3, 98.7-116.0)	113.2 ± 14.2 (113.4, 101.8-121.3)	125.6 ± 22.6 (122.3, 107.8-142.2)	<0.001
Weight (kg)	18.3 ± 5.8 (17.1, 15.0-20.4)	21.1 ± 6.4 (19.8, 16.3-25.0)	29.5 ± 14.6 (25.5, 17.8-38.4)	<0.001
BMI (kg/m^2)	15.3 ± 1.4 (15.3, 14.4-16.3)	16.2 ± 2.2 (15.7, 14.9-16.8)	17.5 ± 2.8 (16.8, 15.4-19.1)	<0.001
Endocrine findings				
T3 (ng/dL)	144.3 ± 26.1 (145.0, 124.0-163.0)[3]	139.2 ± 23.9 (136.5, 122.5-153.8)	136.8 ± 23.6 (136.0, 121.8-152.5)[4]	0.19
T4 (μg/dL)	9.5 ± 1.6 (9.8, 8.4-10.7)[3]	9.6 ± 1.4 (9.6, 8.6-10.7)	9.4 ± 1.7 (9.6, 8.5-10.8)[4]	0.90
FT4 (ng/dL)	1.2 ± 0.1 (1.2, 1.1-1.3)[3]	1.2 ± 0.1 (1.2, 1.1-1.3)	1.2 ± 0.2 (1.2, 1.1-1.3)[4]	0.17
TSH (μU/mL)	2.5 ± 1.1 (2.3, 1.7-3.1)[3]	2.3 ± 1.2 (1.9, 1.4-3.1)	2.4 ± 1.1 (2.3, 1.6-2.9)[4]	0.41
	N=72	N=51	N=80	
Urinary metabolites				
MEHP (μg/g Cr)	54.0 ± 56.4 (38.0, 20.0-80.7)	42.5 ± 47.9 (25.0, 9.6-61.9)	41.8 ± 37.7 (34.1, 18.0-50.9)	0.19
MEOHP (μg/g Cr)	170.7 ± 142.2 (135.7, 83.1-184.6)	146.1 ± 119.8 (115.7,82.6-173.4)	125.8 ± 123.7 (104.7, 71.6-148.1)	0.03
MEHHP (μg/g Cr)	246.6 ± 247.0 (190.3, 115.3-259.1)	197.3 ±136.7 (164.0, 116.5-243.2)	183.2 ± 172.3 (154.5, 98.5-221.8)	0.08
DEHP intake estimated by creatinine excretion-based model (μg/kg/day)	6.6 ± 6.0 (5.0, 3.2-7.8)	5.3 ± 3.7 (4.3, 3.3-6.7)	4.9 ± 4.7 (3.9, 2.5-5.5)	0.03

Abbreviations: BMI = body mass index; Cr = creatinine; DDI = daily DEHP intake; DEHP = di-(2-ethylhexyl) phthalate; FT4 = free thyroxine; IQR = interquartile range; MEHP = mono-(2-ethylhexyl)phthalate; MEOHP = mono-(2-ethyl-5-hydroxylhexyl) phthalate; MEHHP = mono-(2-ethyl-5-oxohexyl) phthalate;T3 = triiodothyronine; T4 = thyroxine; TSH = thyroid-stimulating hormone.

[1]Original data before multiple imputation.

[2]Kruskal-Wallis test for continuous variables and Fischer's exact test for category variables.

[3]One missing data.

[4]Two missing data.

Table S2. Relationship between serum thyroid profiles and di-(2-ethylhexyl) phthalate (DEHP) intake estimated by questionnaire and DEHP estimates by creatinine excretion-based model in multivariate linear regression models after multiple imputation.[1]

	Crude					Adjusted Model[2]			
	**N**	**β**	**SE**	***P***	***P****	**β**	**SE**	***P***	***P****
T3									
Daily DEHP intake (DDI) estimated by questionnaire	228	0.06	0.03	0.02	0.08	0.04	0.03	0.13	0.25
DEHP intake estimated by creatinine excretion-based model[3]	220	0.02	0.37	0.95	0.97	-0.08	0.33	0.82	0.99
T4									
Daily DEHP intake (DDI) estimated by questionnaire	228	0.001	0.002	0.74	0.74	-0.002	0.002	0.36	0.47
DEHP intake estimated by creatinine excretion-based model[3]	220	-0.01	0.02	0.73	0.97	-0.02	0.02	0.39	0.78
FT4									
Daily DEHP intake (DDI) estimated by questionnaire	228	0.00008	0	0.62	0.74	0.00005	0	0.73	0.73
DEHP intake estimated by creatinine excretion-based model[3]	220	0.00007	0.002	0.97	0.97	0.00001	0.002	0.99	0.99
TSH									
Daily DEHP intake (DDI) estimated by questionnaire	228	0.002	0.001	0.05	0.11	0.002	0.001	0.10	0.25
DEHP intake estimated by creatinine excretion-based model[3]	220	0.03	0.02	0.14	0.56	0.02	0.02	0.19	0.76

Abbreviations: BMI = body mass index; DDI = daily DEHP intake; DEHP = di-(2-ethylhexyl)phthalate; FT4 = free thyroxine; T3 = triiodothyronine; T4 = thyroxine; TSH = thyroid-stimulating hormone.
[*]Benjamini-Hochberg adjusted P values.
[1]22 participants not exposed to the listed phthalate-tainted foods were excluded.
[2]Adjusting for age, gender, BMI.
[3]Eight missing data.

Table S3. Relationship between serum thyroid profiles and urinary phthalate metabolites in multivariate linear regression models (N = 220).[1]

	**T3**						**T4**						**FT4**						**TSH**					
	**Crude**			**Adjusted[2]**			**Crude**			**Adjusted[2]**			**Crude**			**Adjusted[2]**			**Crude**			**Adjusted[2]**		
	**β (SE)**	**P**	**P***	**β (SE)**	**P**	**P***	**β (SE)**	**P**	**P***	**β (SE)**	**P**	**P***	**β (SE)**	**P**	**P***	**β (SE)**	**P**	**P***	**β (SE)**	**P**	**P***	**β (SE)**	**P**	**P***
MMP	-0.004 (0.01)	0.61	0.70	-0.002 (0.01)	0.76	0.80	0 (0.001)	0.54	0.82	0 (0.001)	0.45	0.60	0.00005 (0)	0.36	0.76	0.00005 (0)	0.33	0.88	0.00009 (0)	0.82	0.82	0.00006 (0)	0.88	0.88
MEP	0.02 (0.01)	0.03	0.23	0.01 (0.01)	0.13	0.80	0 (0)	0.30	0.75	-0.001 (0)	0.16	0.43	0.00004 (0)	0.38	0.76	0.00003 (0)	0.50	0.93	0 (0)	0.33	0.43	0 (0)	0.43	0.57
MnBP	0.01 (0.01)	0.14	0.28	0.004 (0.01)	0.62	0.80	-0.001 (0.001)	0.37	0.75	-0.001 (0.001)	0.16	0.43	0.00009 (0)	0.11	0.43	0 (0)	0.06	0.24	0.001 (0)	0.01	0.06	0.001 (0)	0.02	0.15
MBzP	-0.01 (0.01)	0.25	0.40	-0.01 (0.01)	0.48	0.80	-0.001 0.001)	0.11	0.75	0 (0.001)	0.56	0.64	0.00003 (0)	0.95	0.95	0.00006 (0)	0.91	0.93	0.001 (0)	0.18	0.31	0.001 (0)	0.11	0.44
MEHP	0.01 (0.04)	0.75	0.75	0.01 (0.03)	0.80	0.80	0.002 (0.002)	0.34	0.75	0.002 (0.002)	0.40	0.60	0.00008 (0)	0.70	0.93	0.00008 (0)	0.68	0.93	0.001 (0.002)	0.50	0.57	0.001 (0.002)	0.59	0.68
MEHHP	0.01 (0.01)	0.40	0.53	-0.003 (0.01)	0.75	0.80	0 (0.001)	0.72	0.82	-0.001 (0.001)	0.27	0.53	0.00008 (0)	0.88	0.95	0.00005 (0)	0.93	0.93	0.001 (0)	0.17	0.31	0 (0)	0.32	0.57
MEOHP	0.03 (0.02)	0.09	0.24	0.01 (0.01)	0.67	0.80	0 (0.001)	0.62	0.82	-0.001 (0.001)	0.15	0.43	0.00003 (0)	0.68	0.93	-0.00001 (0)	0.89	0.93	0.001 (0.001)	0.19	0.31	0.001 (0.001)	0.38	0.57
MiBP	0.01 (0.01)	0.09	0.24	0.01 (0.01)	0.28	0.80	0.00002 (0)	0.99	0.99	0.00003 (0)	0.95	0.95	0.00007 (0)	0.09	0.43	0.00008 (0)	0.06	0.24	0 (0)	0.16	0.31	0 (0)	0.20	0.53

Abbreviations: BMI = body mass index; FT4 = free thyroxine; MBzP = mono-benzyl phthalate; MEHHP = mono(2-ethyl-5-hydroxyhexyl)phthalate; MEHP = mono-(2-ethylhexyl)phthalate; MEOHP = mono-(2-ethyl-5-oxohexyl)phthalate; MEP = mono-ethyl phthalate; MMP = mono-methyl phthalate; MiBP = mono-isobutyl phthalate; MnBP = mono-n-butyl phthalate; T3 = triiodothyronine; T4 = thyroxine; TSH = thyroid-stimulating hormone.

*Benjamini-Hochberg adjusted P values.

[1] 22 participants not exposed to the listed phthalate-tainted foods were excluded.

[2] Adjusted for age, gender, and BMI.
